# Surgery

**Published:** 1902-03

**Authors:** J. Lacy Firth


### SURGERY.

**Gastric Hemorrhage.**—As a result of the recent remarkable advances in the surgical treatment of diseases of the stomach, the surgical aspects of gastric hemorrhage seem destined to receive more attention in the future than they have in the past. The subject was ably discussed by A. W. Mayo Robson in the Hunterian Lectures at the Royal College of Surgeons in March, 1900;[1] again in an address at Edinburgh in February, 1901;[2] and again in conjunction with B. G. A. Moynihan in a recently published book.[3] W. L. Rodman, of Philadelphia, discussed the subject before the American Surgical Association in May, 1900;[4] and again before the American Medical Association in June, 1900.[5] It is an abstract of the writings of these surgeons that we give below. All the writers have published tables of cases collected from various sources, in which operations have been undertaken for the purpose of arresting gastric hemorrhage. Other cases have been reported since these tables were drawn up, in this country for example by C. W. Mansell Moullin[6] and H. Brunton Angus,[7] and also abroad.

**Hemorrhage from Gastric and Duodenal Ulcers.**—Gastric ulcer has been estimated by Ewald to afflict 5 per cent of the German community. Hemorrhage has been variously estimated to occur in from 50 per cent. to 80 per cent. of all cases of gastric ulcer, and to be fatal under ordinary medical treatment in from 3 per cent. to 11 per cent. of those cases in which it does occur. Taking the average of the various estimates, viz., 7 per cent., as the probable mortality from hemorrhage, and applying the figures to the 500,000 population of a city like Leeds, as Robson does, it will be seen that 25,000 of the inhabitants will suffer in the course of their lives from gastric ulceration, that 12,500 of them will have hæmatemesis, and that 875 will die from the hemorrhage. These figures show the great importance of the subject under discussion.

The hemorrhage in gastric ulcer may be from capillaries and small arterioles, or from larger veins or arteries such as the splenic or coronary. Where the ulcer has the form of a superficial erosion of the mucous membrane, the "Exulceratio Simplex" of Dieulafoy, the hemorrhage will be of the former capillary or small arteriole type; when the ulcer is of the ordinary deeper kind, which tends to perforate, the "Ulcus

[1] *Lancet*, 1900, i. 679. [2] *Ibid.*, 1901, i. 375.

[3] *Diseases of the Stomach and their Surgical Treatment*, 1901, p. 130.

[4] *Tr. Am. Surg. Ass.*, 1900, xviii. 38. [5] *Philad. M. J.*, 1900, v. 1303.

[6] *Lancet*, 1900, ii. 1125. [7] *Brit. M. J.*, 1901, i. 696.

Simplex" of Cruveilhier, the hemorrhage may be of either type. It is obvious that medical treatment is much more likely to successfully arrest the former variety of hemorrhage than the latter. Records, however,—notably those of M. Savariaud,[1]—show that there is no constant relationship between the size of the vessel or vessels which bleed and the amount of blood lost. Similarly, there is no constant relationship between the size of the vessel and the period of survival after the hemorrhage. Death may be sudden from capillary hemorrhage, or delayed ten days with an opening in the aorta the size of a haricot bean. It is, therefore, usually impossible to do more than make a guess as to the size of the bleeding vessel. In nearly four-fifths of the fatal cases the bleeding is from vessels of rather large size. These vessels are usually only laterally opened by the ulceration, though occasionally they are completely divided. In the former case natural hæmostasis is prevented; for retraction and contraction of the artery cannot take place, and the "whole force of the heart is then exerted on the clot which tends to form during the syncope that usually occurs when a large quantity of blood is suddenly poured out" (Robson). There is every reason to believe, as Rodman says, that the bleeding is from capillaries and small arterioles in a much greater proportion of the non-fatal than of the fatal cases. Perhaps in a majority of the cases which survive the hemorrhage is of the capillary type. If the size of the bleeding vessel could be determined with a great degree of probability from the symptoms, which at present seems impossible, it would become much easier to decide when surgical treatment was called for. Obviously, medical treatment cannot be wisely relied upon to permanently arrest hemorrhage from an ulcerated artery the size of the ulnar or radial.

We have seen that according to an average estimate 7 per cent. of the cases in which hemorrhage occurs in this disease end fatally. In other words, 93 per cent. of the patients who have hemorrhage survive under medical treatment. For this reason all writers on the subject, with the exception of Dieulafoy, recommend medical treatment for the first attack of hæmatemesis. Dieulafoy[2] advises surgical treatment after the first hemorrhage, if as much as half a litre (℥xvij ss.) of blood has been lost; and one of his cases was successfully operated upon by Cazin after the first hemorrhage. Both Robson and Rodman recommend operation if the hemorrhage is repeated a second time. The former recommends it even during the course of the hemorrhage, if the patient can stand it; the latter advises waiting until the bleeding ceases and the patient rallies from the shock. Both writers again recommend operation for the so-called "chronic hemorrhage" in which frequently repeated small hemorrhages occur, tending gradually to cause death. Rodman collected reports of thirty-one cases in which operations

[1] *Thèse de Paris*, 1898. [2] *Presse méd.*, 1898, 31.

were performed for this chronic form of hemorrhage. Six of the patients died, yielding a mortality of 19.3 per cent. In thirty-two cases of operation for acute hæmatemesis there were thirteen deaths, or a mortality rate of 40.6 per cent. There have been other successful operations performed since these statistics were drawn up. The mortality after operation in the acute cases was thus very high; but it is to be remembered in this connection that in all the cases medical treatment was first tried, and that probably none were operated upon too soon, but many too late, for example after the third and fourth hemorrhages.

**The method of operating.**—Rodman gives the following list of operative methods which have been adopted in attempts to arrest gastric hemorrhage: (1) Partial gastrectomy, or pylorectomy; (2) Gastro-enterostomy; (3) Gastrotomy; (4) Excision; (5) Excision of the ulcer, with ligation of the affected artery; (6) Ligation, *en masse*, of the affected mucous membrane; (7) Suture of the ulcer; (8) Cauterization; (9) Pyloroplasty; (10) Gastrorraphy; (11) Ligature of the principal artery; (12) Curettage of the ulcer with or without cauterization. Of these methods those which accomplish complete removal of the ulcer are the ideal ones; for they not only most effectually check further hemorrhage, but prevent the possibility of future perforation or malignant degeneration. For the complete removal of an ulcer it may be necessary to perform pylorectomy or partial gastrectomy. Up to the present the operation most frequently selected in these cases has been that of gastro-enterostomy, which, by the functional rest it secures for the stomach, favours cicatrization of the ulcer. The operation is indicated when for any reason, such as the locality of the ulcer, existence of adhesions, or the state of the patient, the preceding operation is contra-indicated; and also when multiple ulcers are found, which statistics show may be expected in one case in every five; and, again, when careful exploration of the interior of the stomach shows the hemorrhage to be capillary or from small undiscoverable ulcers. Rodman points out that in at least seven cases exploration of the stomach failed to reveal the source of hemorrhage. He also stated that gastro-enterostomy for hemorrhage had been performed thirteen times with three deaths, or a mortality of 23.07 per cent. In twelve cases where the operation was performed for small but frequently repeated hemorrhages there was no death, which is a most encouraging result.

The method of operating by ligaturing the affected mucous membrane *en masse* has been several times successful. If all the coats of the stomach are included in the ligature, which may be effected by previously pushing or pulling them into a conical eminence, there will be danger of perforation unless supporting Lembert sutures are placed in the serous coat externally. It would appear to be better, therefore, if possible, not to include the serous coat in the ligature. If the ulcer is treated by cauterization, gastro-enterostomy should also be performed. Pyloroplasty is not to be recommended, unless at the same time the ulcer can be completely excised; for if this is not done, stenosis of the pylorus is likely to develop subsequently (Robson). Gastrorraphy is not to be recommended. The hemorrhage of duodenal ulcer, often indistinguishable from that of gastric ulcer, should be treated on the same principles; and when exploration of the stomach for hemorrhage yields negative results, the duodenum should be explored as thoroughly as possible.

Mansell Moullin [1] treated two cases of gastric hemorrhage successfully with "silk ligatures passed in two directions underneath these ulcers by means of curved needles and tied so as to strangulate the base;" and a third case by ligaturing the whole thickness of the stomach wall, and reinforcing with Lembert sutures externally at the puckered spot. Brunton Angus [2] gained a success by passing a purse-string suture around the ulcer and tying it tightly.

The conclusions of the writers we have quoted may be summarized as follows: Operation is strongly indicated (1) in recurring or so-called chronic hæmatemesis from gastric ulcer; (2) in acute hæmatemesis from the same disease, if there is reason to believe that a large vessel has been eroded, or if there has been a second severe hemorrhage; and (3) that usually the best operation is one of three: viz., (*a*) clean excision of the ulcer, which may involve pylorectomy or partial gastrectomy; or (*b*) gastro-enterostomy; or (*c*) ligation, *en masse*, of the affected portion of mucous membrane.

**Hemorrhage from Gastric Carcinoma.**—No operation appears to have been performed in gastric carcinoma for the sole purpose of arresting hemorrhage. Should it be called for, gastrectomy, partial or complete, or gastro-enterostomy would be the operations of choice.

**Hemorrhage in Cirrhosis of the Liver.**—Rodman holds that although no operation appears as yet to have been deliberately performed for gastric hemorrhage considered to be due to cirrhosis of the liver, yet surgical intervention, with limitations, may properly be considered. The results are less likely to be favourable than in the case of gastric ulcer, for there exists a general hemorrhagic tendency in the disease which renders bleeding difficult to arrest in any situation. The conclusion of Preble of Chicago,[3] derived from a study of sixty fatal cases of this form of hemorrhage, are pertinent to the question of surgical intervention. That author found: (1) that gastro-intestinal hemorrhage is not a rare complication of cirrhosis of the liver; (2) that in a third of the cases the first hemorrhage is fatal, and in the remaining two-thirds the hemorrhages continue at intervals for a period lasting from a few months to several years;

[1] *Loc. cit.* [2] *Loc. cit.* [3] *Am. J. M. Sc.*, 1900, cxix. 263.

(3) that in one-third of the cases the diagnosis can be made at or before the first hemorrhage, but in the other cases the diagnosis cannot be made at all, or only after an interval of months or years, during which other symptoms of cirrhosis develop; (4) that œsophageal varices are present in 80 per cent. of the cases, and in half of these the varices show microscopical ruptures, and probably many other ruptures would be found if the veins were tested with air or water; (5) that fatal cases occur in which no œsophageal varices are present, probably from rupture of capillaries in the gastro-intestinal mucous membrane; and (6) that in only 6 per cent. of the cases showing varices are the symptoms of cirrhosis of the liver typical. Mistakes, therefore, have been made in the past and will continue to be made in attempting to distinguish between gastric hemorrhage due to cirrhosis of the liver, and that due to ordinary gastric ulcer; and no doubt some of the cases operated upon for gastric hemorrhage, supposed to be due to ulcer, were in reality cases of venous hemorrhage secondary to cirrhosis of the liver.

**Post-operative Hematemesis.** — Robson reports that he has seen several cases of this form of gastric hemorrhage, and that three of them were fatal. They followed operation for intestinal obstruction, tuberculous peritonitis, hernia, ovarian tumour, and disease of the gall-bladder. Other surgeons have also reported cases, including Von Eiselsberg (six cases), Mansell Moullin (one case), and Winslow (one case). In some of the cases hemorrhages into the gastric mucosa have been found, and in others numerous small ulcers. The cause remains obscure. As individual cases have occurred without general anæsthesia, or sepsis, or post-operative vomiting, or injury of the omentum, no one of these supposed causes will explain every case. Robson, therefore suggests that a reflex nervous disturbance is the cause. Two cases have been operated upon for this form of hemorrhage by Reichard. They ended fatally. Under general medical treatment more cases have recovered than have died, and this form of treatment seems to be indicated.

Here we may allude to the treatment of gastric hemorrhage of all varieties by the administration of copious hot-water enemas, as advocated by Tripier.[1] In several severe cases of hemorrhage, which had resisted other forms of treatment, that writer obtained excellent results by injecting water at a temperature of 112° F. to 120° F., and repeating the injections twice or thrice daily. The injections appear to act reflexly. As Rodman points out, the injections will also tend to combat the shock due to the hemorrhage, and that for this purpose salt may be added in proportion to make normal salt solution.

**Vicarious Hematemesis at the Menstrual Epoch.**—It is open to question whether the reported cases of this affection were not really cases of latent gastric ulcer, or at least that the hemorrhage was due to some other cause than absence of menstruation.

[1] *Semaine méd.*, 1898, xviii. 241–245.

Rodman wrote to fifty prominent gynæcologists and surgeons to learn if they had seen any cases of this kind after removal of the appendages or uterus, or both. Only two reported affirmatively. They had each seen a single case. But if vicarious menstruation of this kind ever occurs, it should occur after the above operations carried out in young women. Robson quotes Petersen's experience of three cases treated surgically. They all died.

J. LACY FIRTH.

